# Cdk2 suppresses IL‐23 expression and the onset of severe acute pancreatitis

**DOI:** 10.1002/iid3.631

**Published:** 2022-05-19

**Authors:** Yanpeng Ma, Longlong Liu, Bin Li, Wenyao Wang, Tingting Zhao

**Affiliations:** ^1^ Department of General Surgery of East District The Second Hospital of Hebei Medical University Shijiazhuang Hebei China; ^2^ Preventive Health Service The Second Hospital of Hebei Medical University Shijiazhuang Hebei China

**Keywords:** acute pancreatitis, CDK, DCAF2, IL‐23, macrophages

## Abstract

**Background:**

Acute pancreatitis is a sudden inflammation of the pancreas. Although interleukin‐23 (IL‐23) is associated with the severity of acute pancreatitis, the underlying mechanism remains largely unknown. Herein, its regulatory mechanisms were explored in this study.

**Methods:**

RNA‐sequencing analysis selected the differently expressed genes in cerulean‐induced acute pancreatitis mice. Polymerase chain reaction analysis determined IL‐23 expression in cyclin‐dependent kinase 2 (Cdk2) short hairpin RNA (shRNA)‐pretreated or DDB1–cullin‐4‐associated factor‐2 (DCAF2)‐overexpressed RAW264.7 cells or CDKs inhibitor AT7519/cullin ring‐finger ubiquitin ligase inhibitor MLN4924‐treated bone marrow‐derived macrophages in the presence of lipopolysaccharides (LPS). Pancreatic damages were evaluated in AT7519‐treated pancreatitis mice.

**Results:**

Pancreatitis mice displayed an increased expression on IL‐23 and a decreased expression of Cdk2. Inhibiting Cdk2 by shRNA or AT7519 significantly induced IL‐23 expression in LPS‐treated RAW cells. Moreover, AT7519 treatment significantly aggravated the severity of acute pancreatitis in mice. Furthermore, AT7519 remarkably increased DCAF2 expression, which was also induced by MLN4924 no matter with or without AT7519 in vitro. On the contrary, overexpressing DCAF2 blocked the stimulatory effect of AT7519 on IL‐23 expression.

**Conclusion:**

Cdk2 negatively regulates IL‐23 expression by inhibiting DCAF2 in acute pancreatitis, indicating that Cdk2 might serve as a promising therapeutic target for acute pancreatitis.

## Graphical abstract

Cyclin‐dependent kinases (CDKs) inhibitor AT7519 aggravated acute pancreatitis in mice. CDKs inhibitor AT7519 suppressed interleukin‐23 (IL‐23) expression by regulating DDB1–cullin‐4‐associated factor‐2 expression.

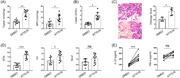

## 1 INTRODUCTION

Pancreatitis is a condition characterized by the inflammation of the pancreas due to the self‐digesting effect of digestive enzymes produced by the pancreas.^1^ Pancreatic damages occur including edema, congestion, bleeding, and necrosis, leading to the symptoms of abdominal pain, bloating, nausea, vomiting, fever, and other symptoms in the patients.^2^ There are two major types of pancreatitis: acute and chronic pancreatitis. Acute pancreatitis is a sudden inflammation of pancreatitis, Macrophages are one of the immune cells that first respond to chemokines released by damaged pancreatic acinar cells during pancreatitis.^3^ In the initial stage of acute pancreatitis, there are local aseptic inflammation and necrosis of acinar cells. Moreover, macrophages differentiate into M1 phenotype in damage‐associated molecular patterns, release proinflammatory cytokines, such as tumor necrosis factor α (TNF‐α), interleukin‐6 (IL‐6), and interleukin‐23 (IL‐23), aggravate the local and systemic inflammation of the pancreas and eventually cause systemic inflammatory response syndrome.^4^

As a new member of the IL‐12 family of heterodimer cytokines, it is composed of the IL‐23A (IL‐23p19) subunit and an IL‐12B (IL‐12/IL‐23p40) subunit, which is shared with IL‐12. IL‐23 exerts biological function only when IL‐23A and IL‐12B bind to form a homodimer and interact with its receptor to activate the downstream signaling pathways. IL‐23 is largely produced by activated dendritic cells, macrophages, and monocytes, and plays a crucial role in the proliferation and stabilization of Th17 cells as well as promoting Th17 cells to produce IL‐17A, IL‐17F, IL‐22, and other cytokines. These inflammatory factors cause the activation and excessive proliferation of keratinocytes, which recruit and activate immune cells through the production of many cytokines, chemokines, and antimicrobial peptides, forming a cascade of immune responses and causing psoriasis damage. Although IL‐23 was identified to be associated with the severity of acute pancreatitis in the patients,^5^ the regulatory mechanism of IL‐23 in acute pancreatitis is still unclear. Therefore, our study aimed to explore the detailed mechanism of IL‐23 in acute pancreatitis.

## 2 MATERIALS AND METHODS

### 2.1 Patients' samples

Blood samples were collected from healthy donors or patients with acute pancreatitis, then peripheral blood mononuclear cells (PBMCs) were isolated from patients' blood. All the procedures were approved by the Second Hospital of Hebei Medical University.

### 2.2 RNA sequencing

Pancreatic tissues collected from mice were lysed in TRIzol buffer (Thermo Fisher Scientific) to extract total RNA and subjected to RNA‐sequencing (RNA‐seq) analysis. RNA integrity number (RIN) > 7.0 was set as the cutoff of sample inclusion for downstream processing by RNA‐seq analysis. RIN is an RNA integrity number to assess integrity values to RNA measurements as well as levels of degradation and fragmentation established by Agilent Technologies. RNA‐seq was performed on the Illumina HiSeq 2500 using sequencing reagents and flow cells, providing up to 300 GB of sequence information per flow cell. The raw reads were mapped to the mm10 reference genome (build mm10), using Bowtie. Gene expressions were quantified by the RSEM software.

### 2.3 Quantitative reverse transcription‐polymerase chain reaction

Total RNA was extracted from 25 to 100 mg of pancreatic tissues or 2 × 10^6^ cells using TRIzol Reagent (Thermo Fisher Scientific). Complementary DNA(cDNA) was synthesized using First‐strand cDNA Synthesis Kit and used as a template to perform quantitative polymerase chain reaction (qPCR) as previously described.^6^ Predesigned Taq‐man probe‐based primers were purchased from IDT. The sequences of qPCR primers were as follows:

Ataxia‐telangiectasia mutated (ATM), cyclin‐dependent kinase 2 (Cdk2), checkpoint kinase 2 (Chk2), Tnf, nitric oxide synthase 2 (Nos2), and DDB1–cullin 4‐associated factor 2 (DCAF2).

Mouse *Il23a* sense 5′‐CATGCTAGCCTGGAACGCACAT‐3′; Mouse *Il23a* antisense 5′‐ACTGGCTGTTGTCCTTGAGTCC‐3′; Mouse *Il17a* sense 5′‐CAGACTACCTCAACCGTTCCAC‐3′; Mouse *Il17a* antisense 5′‐TCCAGCTTTCCCTCCGCATTG‐3′; Mouse *Il17f* sense 5′‐AACCAGGGCATTTCTGTCCCAC‐3′; Mouse *Il17f* antisense 5′‐GGCATTGATGCAGCCTGAGTGT‐3′; Mouse *ATM* sense 5′‐CCAAGATGGCAGTGAACCAGAC‐3′; Mouse *ATM* antisense 5′‐ATGCTGGACAGCTATGGTGGAG‐3′; Mouse *Cdk2* sense 5′‐TCATGGATGCCTCTGCTCTCAC‐3′; Mouse *Cdk2* antisense 5′‐TGAAGGACACGGTGAGAATGGC‐3′; Mouse *Chk2* sense 5′‐GAGGTTCTTGTCTCCAACGGGA‐3′; Mouse *Chk2* antisense 5′‐ATCCTTCAGGGACACTTGGGTC‐3′; Mouse *Tnf* sense 5′‐GGTGCCTATGTCTCAGCCTCTT‐3′; Mouse *Tnf* antisense 5′‐GCCATAGAACTGATGAGAGGGAG‐3′; Mouse *Nos2* sense 5′‐GAGACAGGGAAGTCTGAAGCAC‐3′; Mouse *Nos2* antisense 5′‐CCAGCAGTAGTTGCTCCTCTTCG‐3′; Mouse *Dcaf2* sense 5′‐GTCTGTGTGCTGGTGTCCATCA‐3′; Mouse *Dcaf2* antisense 5′‐CTTCTGAGAGGTCCAACCCACT‐3′.

### 2.4 Cell culture

RAW264.7 cells were cultured in Dulbecco's modified Eagle's medium containing 10% fetal bovine serum (FBS; Gibco). RAW264.7 cells were infected with retrovirus carrying Cdk2 short hairpin RNA (shRNA) for 72 h. Cdk2‐silencing RAW264.7 cells were treated with lipopolysaccharides (LPS) from *Salmonella enterica* serotype enteritidis (100 ng/ml) (L7770; protein content ≤1%, Sigma‐Aldrich) for 2 h, then cells were collected for PCR or Western blot analysis.

To overexpress DCAF2, RAW264.7 cells were infected with retrovirus carrying DCAF2 expressing sequence for 72 h, then treated with AT7519 (50 nM) for 24 h. Cells were collected for PCR or Western blot analysis.

### 2.5 Bone marrow‐derived macrophages

Bone marrow‐derived macrophages (BMDMs) were extracted from the bone of C57BL/6 male mice. After centrifugation at 200*g* for 5 min, cells were collected and resuspended in the medium containing 10% FBS and 50 ng/ml macrophage colony‐stimulating factor for 5 days to get mature macrophages. After pretreated with AT7519 (50 nM) (Selleck Chemicals) with or without MLN4924 (5 nM) (Sigma‐Aldrich) for 24 h, these BMDMs were stimulated with LPS (100 ng/ml) for 2 h.

### 2.6 Mouse model of severe acute pancreatitis

C57BL/6 mice (GemPharmatech) were housed in standard cages at 25°C with free access to water and a commercial rodent diet under pathogen‐free conditions. All experiments were performed in accordance with an experimental guideline approved by the Animal Care and Use Committee of the Second Hospital of Hebei Medical University.

Eight‐week‐old female C57BL/6 mice were intraperitoneally injected with 50 mg/kg cerulean (Sigma‐Aldrich) dissolved in saline to induce severe acute pancreatitis. Untreated mice were intraperitoneally injected with the same volumes of saline as the control. Pancreatic samples were collected from mice 12 h after a single injection of cerulean and cut into two parts. One was stored at −80°C and the other was fixed in 10% formaldehyde for histological analysis.

After pretreated with AT7519 (15 mg/kg) or dimethyl sulfoxide (DMSO; Sigma‐Aldrich), mice were intraperitoneally injected with 50 mg/kg cerulean. Then, mice were killed 12 h after cerulean injection.

### 2.7 Enzyme‐linked immunosorbent assay

The levels of trypsin or myeloperoxidase (MPO) in pancreatic tissue from mice were measured using a coupled Enzymatic Activity Assay Kit (Boster). Lipase levels in the serum were determined by the Activity Assay Kit (R&D Systems) according to the manufacturer's instructions. IL‐23 and TNF‐α levels were measured using corresponding Enzyme‐Linked Immunosorbent Assay Kits (R&D Systems).

### 2.8 Western blot

Total protein was extracted from treated cells using a radioimmunoprecipitation buffer containing with cocktail inhibitor, and a Western blot was performed as previously described.^7^ β‐actin was used as a loading control. Primary antibodies used were β‐actin (A2228; Sigma‐Aldrich), CDK2 (18048; Cell signaling Technology), and DCAF2 (ab72264; Abcam).

### 2.9 Histological analysis

Dissected pancreatic tissues were fixed with formalin, embedded in paraffin, and sectioned at 5–6 mm thickness. After deparaffinized and dehydrated, sections were sequentially stained with hematoxylin and eosin (H&E) using standard laboratory procedures. All tissue sections were assessed under optical light microscope and were taken at 10 random fields (×200 magnification). Pancreatic tissue damage was semiquantitatively graded for edema, acinar necrosis, and inflammatory cell infiltration. Histopathological changes in the pancreas were scored using the pathological score system of Schmidt.

### 2.10 Statistical analysis

The statistical significance of differences between groups was assessed using the GraphPad Prism 5 software. The unpaired two‐tailed Student's *t*‐tests were used for the comparison between groups. The significance of differences was set as *p < .05. Data were shown as means ± standard deviation based on three independent experiments at least.

## 3 RESULTS

### 3.1 Cell cycle‐related molecules were negatively associated with the onset of acute pancreatitis

**FIGURE 1:**
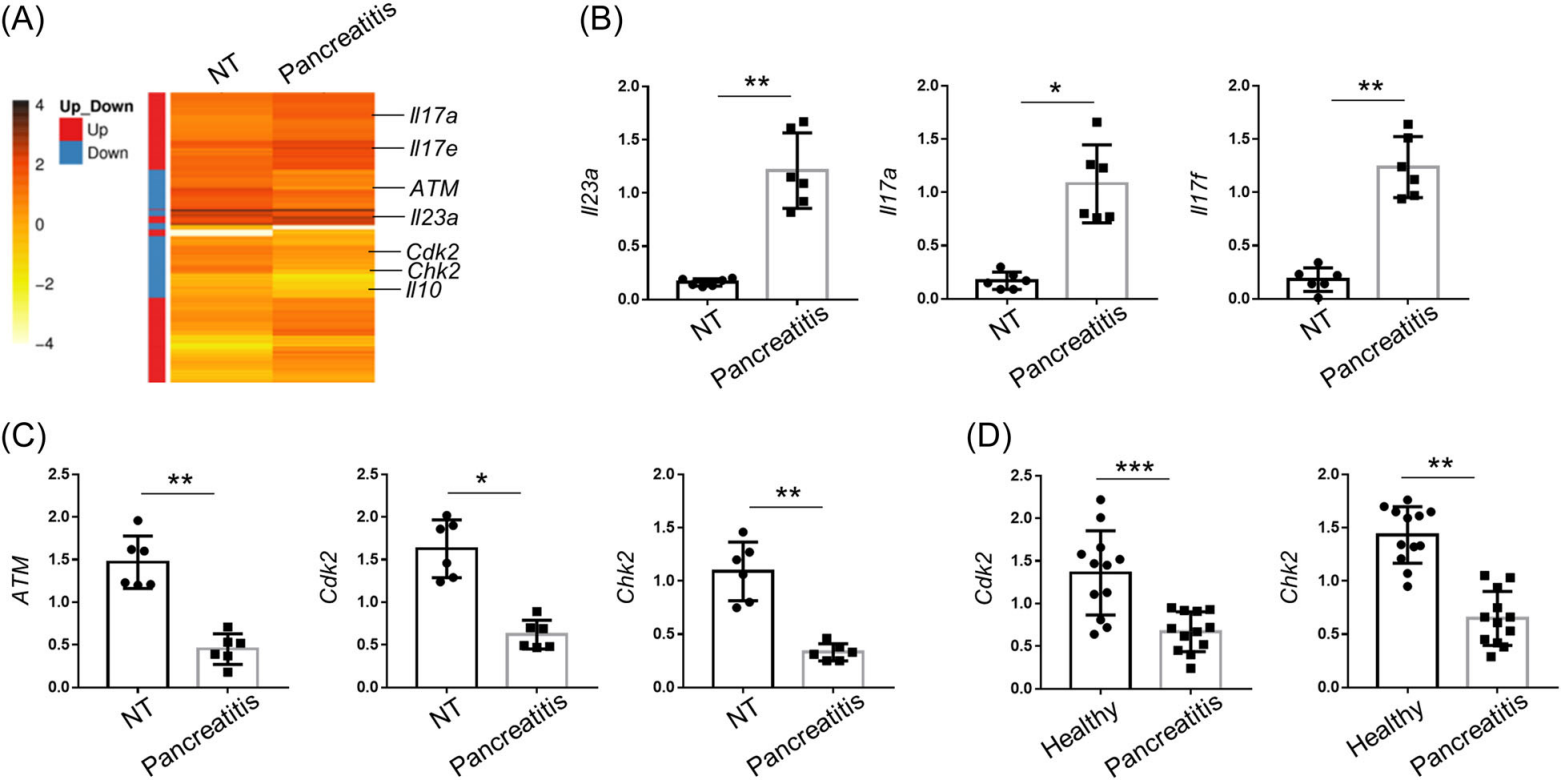
Cell cycle‐related molecules were negatively associated with the onset of acute pancreatitis. (A) Heatmap graphs showing the differentially expressed genes between nontreated and pancreatitis mice. (B,C) qPCR analysis of indicated proinflammatory cytokines and cell cycle‐related genes in the pancreas from nontreated and pancreatitis mice. Actin was used as a loading control and for the relative normalization. (D) qPCR analysis of Cdk2 and Chk2 in the PBMCs from the healthy donors or patients with acute pancreatitis. Data were shown as means ± SD based on three independent experiments at least. Two‐tailed Student's *t*‐tests were performed. ATM, ataxia‐telangiectasia mutated; Cdk2, cyclin‐dependent kinase 2; Chk2, checkpoint kinase 2; NT, untreated; PBMC, peripheral blood mononuclear cell; qPCR, quantitative polymerase chain reaction. *p < .05, **p < .01, and ***p < .005.

To explore the potential pathogenic factors and their regulatory mechanisms during pancreatitis, we collected pancreatic tissues from five untreated or acute pancreatic mice. RNA‐seq data discovered that compared to untreated mice, IL‐23a, IL‐17a, and IL‐17f were remarkably increased while cell cycle‐related molecules, including ATM, Cdk2, and Chk2, were decreased in the mice with acute pancreatitis (Figure 1A). qPCR data also confirmed the upregulated levels of IL‐23a, IL‐17a, and IL‐17f and the downregulated levels of ATM, Cdk2, and Chk2 in the pancreatic tissues of mice with acute pancreatitis (Figures 1B,C). We further collected and separated monocytes from the peripheral blood of 12 patients with acute pancreatitis. Cdk2 and Chk2 expressions were reduced in the PBMC of patients with acute pancreatitis in comparison with the ones from healthy donors (Figure 1D). Thus, cell cycle‐related molecules were negatively associated with the onset of acute pancreatitis.

### 3.2 Cdk2 negatively regulated the expression of IL‐23

**FIGURE 2:**
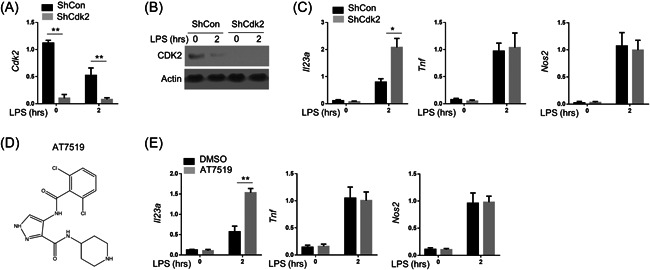
Cdk2 specifically inhibited the expression of IL‐23. RAW264.7 cells were infected with retrovirus carrying Cdk2 shRNA for 72 h. (A,B) qPCR and Western blot assay for the mRNA and protein levels of Cdk2, respectively. (C) Cdk2‐silencing RAW264.7 cells were treated with LPS (100 ng/ml) for 2 h, and inductions of multiple proinflammatory cytokines were measured by qPCR. Actin was used as loading control and for the relative normalization. (D) The chemical structure of CDKs inhibitors, AT7519. (E) BMDMs were generated from the WT B6 mice with M‐CSF (50 ng/ml) for 5 days. After pretreated with AT7519 (50 nM) for 24 h, these BMDMs were stimulated with LPS (100 ng/ml) for 2 h, and inductions of multiple proinflammatory cytokines were measured by qPCR. Data were shown as means ± SD based on three independent experiments at least. Two‐tailed Student's *t*‐tests were performed. BMDM, bone marrow‐derived macrophages; CDK, cyclin‐dependent kinase 2; DMSO, dimethyl sulfoxide; IL‐23, interleukin‐23; LPS, lipopolysaccharides; M‐CSF, macrophage colony‐stimulating factor; mRNA, messneger RNA; Nos2, nitric oxide synthase 2; qPCR, quantitative polymerase chain reaction; shRNA, short hairpin RNA; Tnf, tumor necrosis factor; WT, wild‐type. *p < .05 and **p < .01.

To further evaluate the role of cell cycle‐related molecules during acute pancreatitis, we knockdown Cdk2 in the RAW264.7 cells, which was confirmed by qPCR (Figure 2A) and Western blot (Figure 2B). The expression of IL‐23 was induced by LPS and further increased after knocking down Cdk2 in the RAW cells (Figure 2C). At the same time, Cdk2 knockdown could not further increase LPS‐induced expressions of Tnf and Nos2 (Figure 2C). AT7519 is an ATP‐competitive Cdks inhibitor and has antiproliferative activities on a variety of human tumor cell lines. The structure of AT7519 was shown in Figure 2D. IL‐23 expression was also specifically upregulated in AT7519‐treated BMDMs under the stimulation of LPS (Figure 2E). Correspondingly, the messenger RNA (mRNA) levels of Tnf and Nos2 did not alter by AT7519 in the LPS‐treated BMDMs. Therefore, Cdk2 negatively regulated the expression of IL‐23.

### 3.3 CDKs inhibitor promoted the onset of acute pancreatitis in mice

**FIGURE 3:**
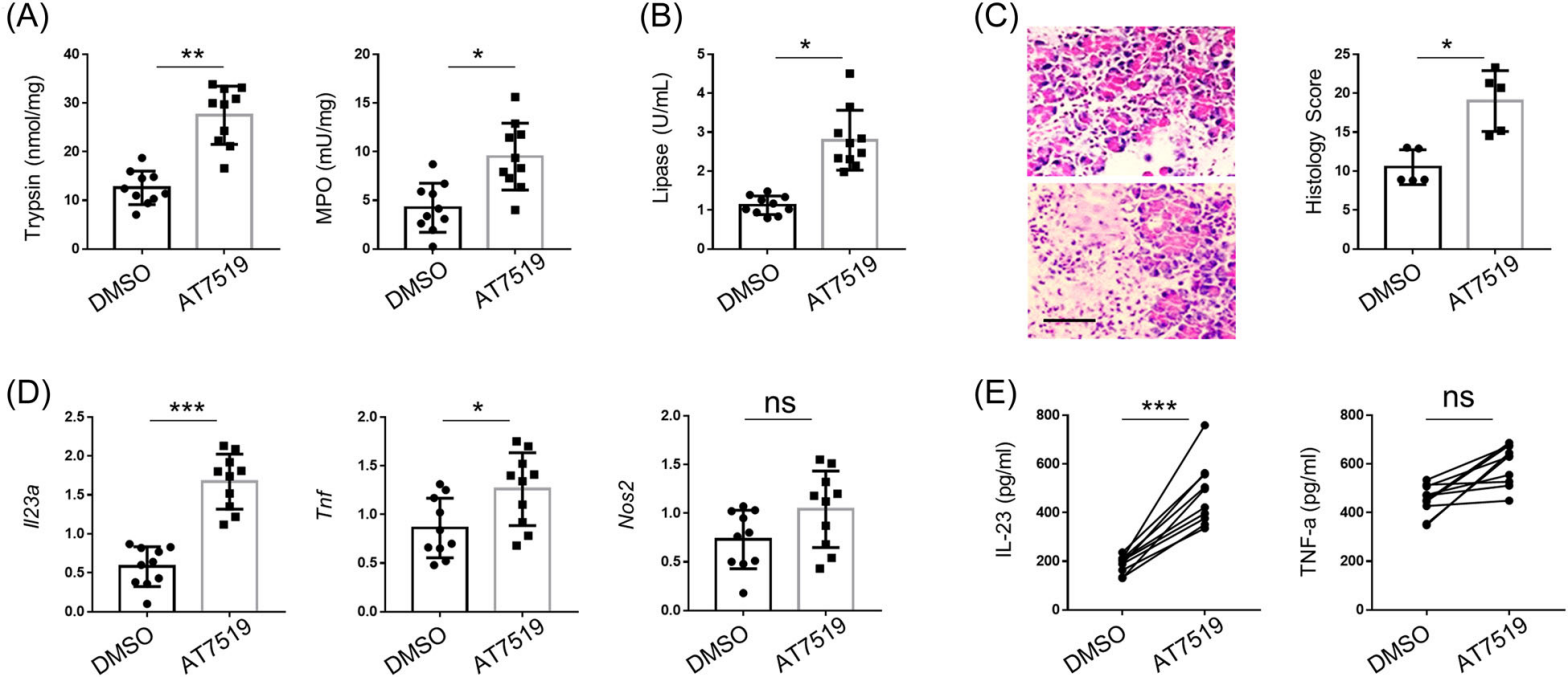
CDKs inhibitor promoted the onset of acute pancreatitis in mice. (A) The levels of trypsin and MPO in nontreated or pancreatitis mice were measured by Activity Assay Kit. (B) The levels of lipase in the serum of nontreated or pancreatitis mice by using a coupled enzyme reaction with Activity Assay Kit. (C) The sections of H&E staining and histology scores of pancreatic tissues. Scale bar = 50 μm. (D) qPCR analysis of indicated proinflammatory cytokines in the pancreas from nontreated and pancreatitis mice. Actin was used as loading control and for the relative normalization. (E) ELISA assay for detecting the protein levels of IL‐23 and TNF‐α in the serum of nontreated and pancreatitis mice. Data were shown as means ± SD based on three independent experiments at least. Two‐tailed Student's *t*‐tests were performed. CDK, cyclin‐dependent kinase 2; DMSO, dimethyl sulfoxide; ELISA, enzyme‐linked immunosorbent assay; H&E, hematoxylin and eosin; MPO, myeloperoxidase IL‐23, interleukin‐23; ns, not significant; Nos2, nitric oxide synthase 2; qPCR, quantitative polymerase chain reaction; TNF‐α, tumor necrosis factor α. *p < .05, **p < .01, and ***p < .005.

Compared to DMSO‐treated pancreatitis mice, the injection of AT7519 significantly induced the levels of pancreatic enzymes in the pancreatic tissues, including trypsin and MPO (Figure 3A) as well as the levels of lipase in the serum (Figure 3B). H&E staining also indicated that CDKs inhibitor AT7519 dramatically aggravated acute pancreatitis in mice (Figure 3C). The mRNA levels of proinflammatory cytokines, including IL‐23a and Tnf, were significantly increased by AT7519 in cerulean‐induced pancreatitis mice (Figure 3D). However, only IL‐23 expression was dramatically increased by AT7519 with no alteration of TNF‐α (Figure 3E). Thereby, CDKs inhibitor AT7519 aggravated acute pancreatitis in mice.

### 3.4 CDKs inhibitor suppressed IL‐23 expression via regulating DCAF2 expression

**FIGURE 4:**
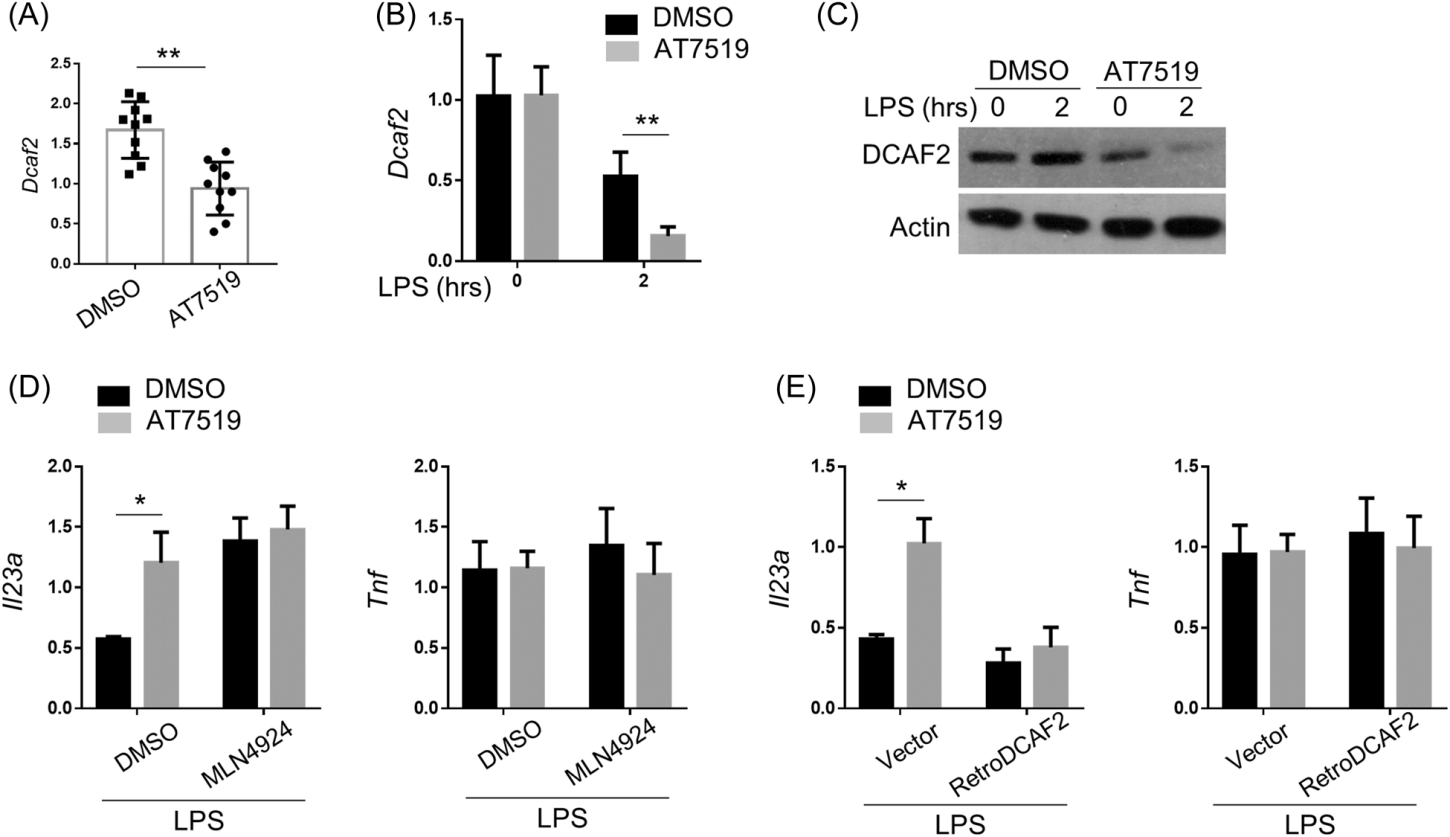
CDKs inhibitor suppressed IL‐23 expression via regulating DCAF2 expression. (A) qPCR analysis of Dcaf2 in the pancreas from nontreated and pancreatitis mice. (B,C) BMDMs were generated from the WT B6 mice with M‐CSF (50 ng/ml) for 5 days. After pretreated with AT7519 (50 nM) for 24 h, these BMDMs were stimulated with LPS (100 ng/ml) for 2 h, and expression of Dcaf2 was measured by qPCR and Western blot. Actin was used as loading control and for the relative normalization. (D) BMDMs were pretreated with MLN4924 (5 nM) and AT7519 (50 nM) for 24 h. The expressions of Il23a and Tnf were evaluated by qPCR. (E) RAW264.7 cells were infected with retrovirus carrying DCAF2 expressing sequence and incubated with AT7519 (50 nM) for 24 h. The inductions of Il23a and Tnf were evaluated by qPCR. Data were shown as means ± SD based on three independent experiments at least. Two‐tailed Student *t*‐tests were performed. BMDM, bone marrow‐derived macrophages; CDK, cyclin‐dependent kinase 2; DCAF2, DDB1–cullin‐4‐associated factor‐2; DMSO, dimethyl sulfoxide; IL‐23, interleukin‐23; LPS, lipopolysaccharides; M‐CSF, macrophage colony‐stimulating factor; qPCR, quantitative polymerase chain reaction; Tnf, tumor necrosis factor; WT, wild‐type. *p < .05 and **p < .01.

The previous publication indicated that the deletion of cell cycle‐related DCAF2 caused the specifical upregulation of IL‐23^8^; therefore, we first evaluated the regulatory effect of CDKs inhibitor AT7519 on the expression of DCAF2. Compared to DMSO, AT7519 injection partially inhibited the expression of DCAF2 in the pancreatitis mice (Figure 4A). In vitro, LPS reduced DCAF2 expression in RAW cells, and AT7519 further decreased its expression (Figure 4B,C). To verify the function of DCAF2 in this process, we used Cullin ring‐finger ubiquitin ligase‐4 (CRL4) inhibitor MLN4924 to pretreat these cells. IL‐23a expression was significantly induced by MLN4924 and could not be further induced by MLN4924 in AT7519‐pretreated RAW cells (Figure 4D). However, Tnf expression could not be altered by either AT7519 or MLN4924. On the contrary, overexpression of DCAF2 also blocked IL‐23 expression in RAW cells, which was induced by AT7519 in the presence of LPS (Figure 4E). Tnf expression was not altered by overexpression of DCAF2. Altogether, these data suggested that CDKs inhibitor AT7519 suppressed IL‐23 expression by regulating DCAF2 expression.

## 4 DISCUSSION

Acute pancreatitis is an inflammatory condition in the pancreas that is sometimes deadly. Despite the great improvement in clinical care medicine over the last decade, the mortality rate of acute pancreatitis remains at approximately 10%. Diagnosis of pancreatic problems is always difficult because of its relative inaccessibility. Therefore, to develop novel therapeutic strategies, it is necessary to explore the detailed mechanism of acute pancreatitis.

Innate immune receptors, particularly pattern‐recognition receptors (PRRs), recognize a lot of molecular patterns associated with invading pathogens, leading to the production of IL‐23. Then PRRs activate its downstream signaling pathway, including canonical nuclear factor κ‐light‐chain‐enhancer of activated B cells and mitogen‐activated protein kinase (MAPK), which contributes to secretions of proinflammatory cytokines, eventually inflammation.^9^, ^10^, ^11^ Therefore, IL‐23 can be recognized as the biomarker of various inflammation‐related diseases, including acute pancreatitis. Moreover, IL‐23 levels were also associated with the severity of acute pancreatitis.^5^ However, the mechanism of IL‐23 in acute pancreatitis is still largely unclear; therefore, our study aimed to explore the regulatory upstream of IL‐23 in acute pancreatitis, leading to the development of a novel therapeutic approach or candidate drug to treat patients with acute pancreatitis.

Our study first discovered that the expression of IL‐23 was significantly increased by RNA‐seq in mice with severe acute pancreatitis, with a remarkable decrease in the expressions of cell cycle‐related molecules, including ATM, Cdk2, and Chk2. Then, using shRNA or CDKs inhibitor to knockdown Cdk2 could induce the expression of IL‐23, which suggested that Cdk2 negatively regulated IL‐23 expression in LPS‐treated RAW 264.7 cells as well as the severity of pancreatitis. Consistent with a previous study that CRL4^DCAF2^ negatively regulated IL‐23 expression,^8^ the inhibition of Cdk2 also decreased the expression of DCAF2. Moreover, overexpression of DCAF2 could well restore IL‐23 expression induced by the inhibition of Cdk2. The above results indicated that Cdk2 may be one of the potential therapeutic targets for acute pancreatitis.

AT7519 is a multi‐CDK inhibitor for CDK1, 2, 4, 6, and 9 with less potent to CDK3 and little active to CDK7.^12^, ^13^, ^14^ Although AT7519 is not a special inhibitor of Cdk2, our data that AT7519 could dramatically induce the expression of IL‐23, might not clearly conclude that IL‐23 was specially regulated by Cdk2. Therefore, we used retrovirus carrying Cdk2 to knockdown Cdk2 in RAW cells, and IL‐23 was also increased by Cdk2‐shRNA, which got similar results as CDKs inhibitor AT7519. Thereby, our data could conclude that Cdk2 specially inhibited IL‐23 expression.

MLN4924, also known as pevonedistat, is a potent and highly selective small‐molecule neuronal precursor cell‐expressed developmentally downregulated protein 8‐activating enzyme to suppress the entire process of neddylation modification cascade, contributing to inactivation of CRLs, because cullin neddylation is required to activate CRLs.^15^ MLN4924 is a potential candidate drug in Phase 1 of a clinical trial to treat patients with acute myeloid leukemia and myelodysplastic syndromes.^16^ Moreover, MLN4924 has an anticancer activity to serve as a novel approach in cancer therapy.^17^, ^18^, ^19^, ^20^ Although MLN4924 is not a special inhibitor of CRL4, MLN4924 is always indicated to inhibit CRL4 as well as DCAF2, also known as CDT2 or DTL, which is one of the substrate adaptors of CRL4. Till now, there is no publication exploring the role of DCAF2 in acute pancreatitis, and our current project was for the first time exclude the relationship between DCAF2 and acute pancreatitis.

## 5 CONCLUSION

Our data elucidated the regulatory effect of Cdk2 and DCAF2 on IL‐23 expression in acute pancreatitis. These findings suggest that Cdk2 could serve as a potential therapeutic target for acute pancreatitis, and CDKs inhibitor AT7519 or CRLs inhibitor MLN4924 might be applied to treat acute pancreatitis.

## AUTHOR CONTRIBUTIONS

Yanpeng Ma has given substantial contributions to the conception of the design of the manuscript. Longlong Liu, Bin Li, Wenyao Wang, and Tingting Zhao contributed to the acquisition, analysis, and interpretation of the data. All authors have participated in drafting the manuscript, Yanpeng Ma revised it critically. All authors read and approved the final version of the manuscript.

## CONFLICTS OF INTEREST

The authors declare no conflicts of interest.

## Data Availability

Data could be obtained upon reasonable request from the corresponding author.
